# Clinical and Virological Features of Acute Hepatic Exacerbations in Patients With Chronic Hepatitis B Virus Infection

**DOI:** 10.7759/cureus.15937

**Published:** 2021-06-26

**Authors:** Remzi Bestas, Kendal Yalcin

**Affiliations:** 1 Department of Gastroenterology, Memorial Dicle Hospital, Diyarbakir, TUR; 2 Department of Gastroenterology, Dicle University School of Medicine, Diyarbakir, TUR

**Keywords:** chronic hepatitis b infection, hepatic exacerbation, hepatitis b virus, flare, clinic

## Abstract

Background and aim: In this study, we aimed to perform a comprehensive analysis of patients with acute hepatic flares observed during the course of chronic hepatitis B infection in order to provide early diagnosis, management, and best characterization of this unique group of hepatitis B patients.

Patients and methods: The study was designed in a retrospective and prospective manner. Chronic hepatitis B patients with acute hepatic flares, admitted to the Department of Gastroenterology and Hepatology were enrolled in the study. Demographic, clinical, biochemical, and virological findings were recorded via pre-prepared forms.

Results: The study was conducted on 125 patients. The mean age was 34.08 ± 12.68 and the male to female ratio was determined as 2.28. Over 117 patients (93.6%) had at least one symptom. The most common symptoms and signs were fatigue (81.6%), anorexia (64%), jaundice (60%), and nausea (52%). Anti-HBc immunoglobulin M (IgM) antibody was detected in 24 patients (19.2%) and serum hepatitis B virus (HBV) deoxyribonucleic acid (DNA) was positive in 107 (85.6%) patients. The most common cause of exacerbations was spontaneous hepatic flares (80.8%).

Conclusion: According to the results of this single-center study, acute hepatic exacerbations are more common in young men. The disease usually presents with non-specific symptoms and jaundice is the most common finding. As a sign of intensive inflammation and hepatocellular injury, serum ferritin levels seem to be high. Serum HBV DNA and anti-HBc IgM positivity with elevated alpha-fetoprotein (AFP) levels are presenting features of acute hepatic exacerbations.

## Introduction

Although there is no consensus on criteria of definition and diagnosis for acute hepatic exacerbations, it depicts an acute elevation of serum aminotransferases in a patient with chronic liver disease with or without symptoms of hepatitis [1-3]. Acute hepatic flares may be caused by spontaneous reactivation of hepatitis B virus (HBV) infection, exposure to immunosuppressive agents or development of immune deficiency, rebound immune response to HBV following termination of immunosuppressive treatment, structural change in the virus, superinfection with other viruses like hepatitis C virus (HCV), hepatitis D virus (HDV), HIV, and exposure to alcohol and drugs which lead to liver damage [4].

Severe acute exacerbation is a unique presentation of chronic hepatitis B characterized by a very high alanine aminotransferase level accompanied by jaundice and hepatic decompensation [5]. The majority of hepatic flares are caused by an alteration in the balance between the immune response to HBV and viral replication with a variable degree. So, the clinical spectrum of hepatic exacerbation may vary from a fully asymptomatic clinical state to symptoms of typical acute hepatitis as well as to manifestations of acute hepatic decompensation and hepatic failure [6].

Several definitions have been used for alanine aminotransferase (ALT) flares in chronic HBV patients. Generally, a >2 to 5-fold ALT increase compared to baseline values is considered to be sufficient for "reactivation" of hepatitis in a chronic carrier.

This trial aims to evaluate demographic, clinical, biochemical, virological, and etiological characteristics of acute hepatic exacerbations in patients with chronic HBV infection to provide early diagnosis, management, and best characterization of this unique group of HBV patients.

## Materials and methods

Patients and study design

Demographic data, laboratory results, and virological markers of hepatitis B patients with acute hepatic flares referring to Department of Gastroenterology and Hepatology of Dicle University School of Medicine. Demographic data, clinical, biochemical, and virological profiles of acute hepatic flare in patients with chronic HBV infection were analyzed.

Patients with known or established chronic HBV infection who had acute hepatic flares were enrolled into this study. Diagnosis of chronic hepatitis B was based on hepatitis B surface antigen (HBsAg) positivity in serum for a duration of at least six months. Acute hepatic flare was defined as an intermittent elevation of aminotransferase activity over five times the upper limit of normal values or an increase of more than twice the baseline value.

Hepatitis A, C, D, non-hepatotropic viruses, such as herpes simplex virus (HSV), cytomegalovirus (CMV), and Epstein-Barr virus (EBV), drugs, heavy alcohol consumption, herbal medicines, autoimmune hepatitis, hepatic ischemia, and pregnancy-related liver disease were investigated separately in every patient.

Among 129 patients whose files were assessed retrospectively, 72 cases with incomplete demographic data, clinical findings, and laboratory values were excluded from the trial. Additionally, 68 patients with chronic hepatitis B infection, referring to our department with elevated transaminase levels and fulfilling the criteria for acute hepatic flare were enrolled for prospective evaluation. Finally, a total of 125 patients fulfilling the required criteria were enrolled in the trial.

Data collection

Clinical symptoms, findings of physical examination, and laboratory data of patients were collected. Laboratory values were analyzed as a mean of three full blood counts (with ethylenediaminetetraacetic acid (EDTA) tube in CELL-DYN 3700 device with automatic optic laser impedance system). Prothrombin time (PTT) was tested by Siemens brand, CA7000 Sysmex model device with ThromborelS’kit. Laboratory evaluations were performed for glucose, urea, creatinine, ALT, aminotransferase (AST), alkaline phosphatase (ALP), gamma-glutamyltransferase (GGT), lactate dehydrogenase (LDH), creatine kinase (CK), total bilirubin, total protein, and albumin (in gel biochemistry tube with Architect C 1600 and enzymatic method), ferritin (in gel biochemistry tube with Cobase 601 kit and electrochemoluminescence test method), alpha-fetoprotein (AFP; in gel biochemistry tube with Immulite 2000 kit and immunoassay method), anti-nuclear antibody (ANA) and anti-smooth muscle antibody (ASMA; in gel biochemistry tube with ASTRA kit and immunofluorescent method), HSV, CMV, and EBV panel (in gel biochemistry tube with ELFA method).

HBV viral markers were tested by Enzyme Immunoassay (EIA) and HBV-DNA was analyzed by COBAS® AmpliPrep/COBAS® TaqMan® HBV Test version 2 (Roche Diagnostics GmbH, D-68298 Mannheim, Germany) diagnostics. Anti-HCV was tested by second-generation EIA test while total anti-delta was analyzed by EIA.

Imaging assessments of patients with a pre-diagnosis were carried out by a blind investigator with USG (3,5-7,5 probe TOSHIBA SSH-140A USG device).

Demographic data, clinical characteristics, biological, and virological parameters of all patients retrospectively enrolled in the trial were collected via information analyzed with a similar design and recorded in special forms designed in the Department of Gastroenterology and Hepatology, patient files in hospital archive, data in the hospital automation system, and by telephone interviews. Data of patients who were prospectively enrolled in the trial were collected during referral visits.

Statistical evaluation

Descriptive statistics related to continuous variables were indicated as mean and standard deviation (SD) values. Discrete variables were expressed as ratios by conversion into cross tables. Correlations between two variables were calculated by the Pearson Correlation Coefficient method. Chi-square test with Yates correction was used in the analysis of cross tables. Hypotheses were two-way and p≤0.05 was regarded as statistically significant. Statistical analysis was realized by Statistical Product and Service Solutions (SPSS) 16.0 for Windows (SPSS, Inc., Chicago, IL, USA) package program.

## Results

Among 125 patients enrolled in the study, 57 were retrospective cases while 68 patients were followed up prospectively. The mean age of patients was 34.08 ± 12.68, varied between 15 and 76 years. The male to female ratio was 2.28 (87/38). Mean age was determined as 33.37±12.95 years for male patients and as 35.71±12.05 for female patients. The demographic data of enrolled patients are shown in Table 1.

**Table 1:** Demographic characteristics of patients with acute hepatic exacerbations

Variables	Results
Age (mean±SD)	34.08±12.68
Male (n, %)	87 (69.6%)
Female (n, %)	38 (30.4%)
Positive family history of chronic HBV infection (n, %)	58 (46.4%)
Family history of chronic HBV infection negative or unknown (n, %)	67 (53.6%)
Alcohol consumption (n, %)	4 (3.2%)
Exposure to drug-herbal medicines (n, %)	2 (1.6%)
Use of immunosuppressive drugs (n, %)	8 (6.4%)

Clinical features

At least one symptom was found in 117 of 125 patients (93.6%) enrolled in the study while no symptom was found in only eight patients (6.4%). The most common symptoms were designated as malaise, anorexia, and nausea. While abdominal pain, vomiting, and flu-like symptoms were less common, high fever was determined in only one patient. Clinical features of patients are summarized in Table 2.

**Table 2:** Clinical characteristics of patients with acute hepatic exacerbations

Variables	Number of patients (n)	(%)
Malaise-fatigue	102	81.6%
Anorexia	80	64%
Nausea	65	52%
Vomiting	27	21.6%
Abdominal pain	56	44.8%
Flu-like symptoms	11	8.8%
Findings of physical examination		
High fever	1	0.8%
Icterus	75	60%
Hepatomegaly	28	22.4%
Splenomegaly	25	20%
Hepatic encephalopathy	1	0.8%

Biochemical characteristics

ALT levels were determined to be increased more than 10 times in 108 patients (86.4%), 5-10 times in 14 patients (11.2%), and 2-5 times in 3 cases (2.4%). Laboratory data of patients are shown in Table 3. A positive correlation was found between ALT level and bilirubin (p<0.0001) as well as between levels of GGT (p<0.05) and PTT (p=0.006). A negative correlation was determined between AFP and albumin levels (p=0.007).

**Table 3:** Initial laboratory values of patients with acute hepatic exacerbations ALT: alanine aminotransferase, AST: aminotransferase, ALP: alkaline phosphatase, GGT: gamma-glutamyltransferase, LDH: lactate dehydrogenase, AFP: alpha-fetoprotein.

Variables	Results (mean±SD)
White blood cells (K/UL)	6515.20±1967.63
Hemoglobin (g/dl)	13.82±1.63
Platelets (K/UL)	234970.40±118101.67
Prothrombin time (sec)	14.97±5.18
Albumin (g/dl)	3.61±0.68
ALT (U/L)	1094.10±875.13
AST (U/L)	797.56±815.73
Total bilirubin (mg/dl)	6.84±8.47
ALP (U/L)	163.12±104.06
GGT (U/L)	167.19±152.66
LDH (U/L)	423.24±280.94
AFP (IU/ml)	18.79±70.25
Log_10_ HBV-DNA (IU/ml)	5.8±2.07
Log_10_ HBV-DNA (copies/ml)	6.58±2.11

Virological characteristics

Serum HBsAg and anti-HBc IgG were positive in all of the 125 patients. Anti-HBc IgM positivity was found in 24 patients (19.2%). While 62 patients (49.6%) were HBeAg positive, 63 (50.4%) were specified as HBeAg negative. In 18 patients (14.4%), serum HBV DNA was undetectable while positive values of detectable level were found in 107 patients (85.6%). The mean level of HBV DNA Log_10_ was 5.8±2.07 IU/ml and 6.58±2.11 copies/ml. While anti-HCV was negative in all patients, anti-delta was found to be positive in four patients (3.2%) and anti-HAV IgM was positive in two cases (1.6%). Anti-HBc IgM titers showed a positive correlation with biochemical parameters of ALT (p=0.002) and ferritin (p=0.004).

Assessment of Causes Associated With Exacerbations

The causes of exacerbations are shown in Table 4. The number of flares in cirrhotic patients was determined as 9 (7.2%).

**Table 4:** Etiological distribution of acute hepatic exacerbation

Causes of exacerbation	Number of patients (n)	(%)
Spontaneous hepatic flares due to HBV infection	101	80.8
Hepatic flares associated with immunosuppressive treatment	8	6.4
Hepatic flares in patients receiving anti-viral therapy	3	2.4
Hepatic flares due to the withdrawal of treatment	7	5.6
Flares due to HDV infection	4	3.2
Flares due to HAV infection	2	1.6

## Discussion

HBV infection is a significant health issue worldwide and it is estimated that approximately two billion individuals around the world are affected by this disease [7]. Turkey is regarded as a moderately endemic area with a prevalence rate of 2-7% for hepatitis B infection, but the prevalence of hepatitis B varies in different regions. Prevalence increases from west to east, with the highest endemicity observed in South-eastern Turkey, with a rate of HBsAg positivity of up to 10% [8-11].

Acute hepatic exacerbation is a well-known complication of chronic HBV infection. These flares may develop spontaneously and may lead to a misdiagnosis of acute hepatitis B. As per definition, acute hepatic flare indicates an abrupt elevation in serum transaminases in a patient with chronic HBV infection, with or without symptoms of hepatitis. The majority of these flares are caused by an alteration in the balance between immune response against HBV and viral replication [12,13]. The objective of this study is to evaluate demographic, clinical, biochemical, virological characteristics, and etiological causes of acute hepatic flares in patients with chronic HBV infection.

In several of the previous studies, the risk of hepatic exacerbation was shown to be increased in men and in young ages [14,15]. Similarly, in this study, there was a dominance of male patients with a younger age. This finding supports the results of previous studies which indicate that male gender and young age is a risk factor for acute hepatic flares.

The number of patients with a positive family history of hepatitis B was 58 (46.4%). Comparison of our results with previous studies [16,17] revealed a higher rate of family history of hepatitis B infection among our cases. In studies conducted in Turkey, horizontal transmission was reported to be more common, as is the case in other moderately endemic regions [10]. In a study conducted by Han et al. [16], a positive family history of hepatitis B infection was determined as an auxiliary finding in favor of chronic HBV infection for differential diagnosis of acute hepatitis B infection and acute hepatic flares in chronic hepatitis B patients. A high rate of positive family history of hepatitis B among our patients provides further support for these results.

The presence of at least one symptom in 117 of 125 patients (93.6%) shows that majority of patients experience a symptomatic attack during acute hepatic flares. The results of our study show that majority of patients experience mild non-specific symptoms like malaise, fatigue, nausea, vomiting, abdominal pain, and anorexia during acute hepatic flares. On the other hand, it should be kept in mind that clinical manifestations may vary from non-specific symptoms to signs of hepatic failure, with more severe flare attacks in cirrhotic patients [1,4,6]. Comparison of symptoms and rates of symptoms in the current study with previous studies evaluating clinical symptoms in acute flares [16] revealed that our patients were more symptomatic than the cases in other studies.

Assessment of physical examination findings of the current study revealed a significantly higher rate of jaundice in 60% of the patients, indicating that more than half of the cases with acute hepatic flares experience icteric attacks. Besides, hepatomegaly and splenomegaly were found in 22.4% and 20% of our cases, respectively. Comparison of these findings with results of a similar study [16] showed similar rates of jaundice and hepatomegaly while splenomegaly was less common in our patients. The presence of jaundice in more than half of the patients reflects the fact that patients with acute hepatic flares, usually manifesting with nonspecific symptoms where the disease goes undiagnosed, generally refer to hospital during icteric attacks. Therefore, jaundice is a significant finding in terms of early diagnosis and provision of suitable treatment in acute hepatic flares.

Acute hepatic flares may lead to alterations in a number of biochemical parameters, especially in aminotransferase levels. Comparison of our laboratory results with a similar study [16] showed considerable similarity. Results of our study indicate that elevated transaminase levels in acute hepatic flares are generally accompanied by high ALP, GGT, and LDH levels as well as prolonged PTT. As observed by Kumar et al. in a previous study [18], none of these biochemical parameters are suitable for use in the differential diagnosis of acute hepatitis B infection and acute hepatic flare associated with CHB because similar findings are also found in acute hepatitis B infection. Positive correlation between ALT level and bilirubin (p<0.0001), GGT (p<0.05), and PTT (p=0.006) is an expected outcome and indicates an increased rate of jaundice and coagulopathy in parallel to the severity of hepatocellular damage.

Although AFP increases in liver cancers, elevated AFP levels may accompany acute hepatic flare attacks and this may lead to suspected hepatocellular cancer [19,20]. In the current study, elevated levels of AFP were found in a substantial portion of patients with acute hepatic flare (42.4%). This finding indicates that elevated AFP level is a suggestive finding of acute hepatic flares, as reported previously [16]. Moreover, no correlation was found between AFP levels and transaminase, bilirubin, ALP, and HBV DNA titers. These results indicate that elevated AFP in acute hepatic flares is not directly associated with severity of acute hepatocellular damage and viral load and suggests that stage of underlying chronic liver disease or other unknown mechanisms may also play a role in this finding.

Anti-HBc IgM, which is generally used as a diagnostic marker for acute hepatitis B, may also be detected in this unique group of HBV patients [21,22]. In the current trial, anti-HBc IgM positivity was detected in a substantial portion of patients. These findings are in parallel with the results obtained in previous trials [17]. So, it should be kept in mind that the disease may present with the positivity of anti-HBc IgM, as is the case in acute hepatitis B infection [23].

Spontaneous exacerbation of chronic hepatitis B is usually caused by the reactivation of HBV infection. This is characterized by an elevation of serum HBV DNA, followed by an increase in serum aminotransferases. High serum HBV DNA detectability rates with relatively high levels of viral replication were found in our cases. This may reflect an early diagnosis because, in acute hepatic flares, HBV DNA levels start to fall as transaminase levels are elevated and HBV DNA levels decrease to nadir or undetectable levels in patients diagnosed in later stages of the disease [24-26].

In this study, spontaneous hepatic flares were found to be the most common etiological cause of disease activation in patients with chronic HBV infection. Although the majority of acute hepatic flares are cases of spontaneous exacerbations, hepatic flares associated with immunosuppressive treatment, drug withdrawal hepatitis, and acute hepatitis A virus infection are of utmost importance in terms of standing out as preventable causes. Therefore, patients with hepatitis B infection should be immunized with hepatitis A vaccine, and in cases of chronic hepatitis B, should be advised to strictly comply with treatment regimens. Besides, all patients regarded as candidates for immunosuppressive treatment should be investigated for hepatitis B infection and antiviral treatment should be initiated prior to treatment in positive cases. Since exacerbations may also be observed in patients under treatment, regular follow-up is essential.

The limitation of this study is that it is partially retrospective. Therefore, the treatment responses of the patients could not be evaluated. In our study, the age, gender, and laboratory findings of patients with acute hepatic exacerbation were similar to previous studies. However, hepatitis B family history and symptoms were found to be higher in patients compared to similar studies. In addition, spontaneous hepatic flares were found to be the most common etiological cause of disease activation and anti-HBc IgM positivity was detected in a substantial portion of patients. These two findings were parallel with the results of previous studies. In this partially retrospective study, demographic, clinical, biochemical, virological characteristics, and etiological causes of acute hepatic exacerbations in chronic HBV infection were evaluated. We expect these results to contribute to the early diagnosis and initiation of treatment of acute hepatic exacerbations, which can have serious fatal consequences.

## Conclusions

Acute hepatic exacerbations induced by various causes in chronic HBV infection may present with different manifestations, varying from an asymptomatic state to signs and symptoms of liver failure. The disease generally presents with non-specific symptoms and jaundice is the most common sign observed in patients. As per our study results, acute hepatic flares are more common in young men with a positive family history of HBV. In these patients, high ferritin levels are detected as an indicator of hepatocellular damage and an excessive amount of inflammation. Positivity of serum HBV DNA and anti-HBc IgM plus high AFP values are common findings of acute hepatic exacerbations.
